# 

**Published:** 1821-09-01

**Authors:** 


### Contents

OF

### No. 6. Vol. II. for SEPTEMBER, 1821.

Art. I.

Memoires de Chirurgie Militaire et Campagnes, &c. That is, Memoirs of Military Surgery and Campaigns; by Baron Larrey, Vol. IV. containing an Account of the Russian Campaign of 1812, and 1813 - - - - - - - 237

II.

Mr. Henry Jeffreys on the Use of Cubebs in the Cure of Gonorrhœa - - - - - - - - - - - - - - - - 271

III.

1. Transactions of the Physico-Medical Society of New York, Vol. I. - - - - - - - - - - - - - - } 275
2. The Philadelphia Journal of the Medical and Physical Sciences, No. 1. - - - - - - - - - - - - } 275

Art. 1. Gastrotomy - - - - - - - - - - - - - - 278

—— 2. Paralysis - - - - - - - - - - - - - - 279

—— 3. Hepatic Functions - - - - - - - - - - - 280

—— 4. Emetics in Spasmodic Diseases- - - - - - 282

IV.

A Treatise on Indigestion and its Consequences, called Bilious and Nervous Complaints, &c. By Dr. A. P. W. Philip. 284

State of the Physiological Question, respecting Digestion in Animals after the Par Vagum is divided - - - - - 311

V.

A Dictionary of Chemistry, by Andrew Ure, M.D.- - - 315

VI.

Transactions of the Association of Fellows and Licentiates of the King's and Queen's College of Physicians in Ireland, Vol. III. - - - - - - - - - - - - - - - - - 327

Art. 1. Dr. Robert Reid on the Pathology and Treatment of Fever - - - - - - - - - - - 327
—— 2. Dr. Harrison's Case of Hæmorrhage- - - - 334
—— 3. Dr. Robinson's Cases of Eruptive Diseases - - 335
—— 4. Dr. Ireland's Case of Emphysema without external violence - - - - - - - - - - - - 335

VII.

Professor Vacca's Memorial on the Recto-Vesical Method of extracting Urinary Calculi, with Cases - - - - - - 337

VIII.

1. Dr. Thomas on Chronic Affections of the Digestive Organs } 348

2. Article "Clysma," in Dict. des Sciences Med. Vol. V. - } 348

3. Article "Sangsue," in Dict. des Sciences Medicales Vol. XLIX. - - - - - - - - - - - - - - - - - } 348

IX.

Dr. Nicholl on Disordered States of the Cerebral Structures, in Infants - - - - - - - - - - - - - - - 361

X.

Mr. Travers on the Treatment of Diseases of the Eye - - 369

XI.

Dr. John Gordon Smith, on the Principles of Forensic Medicine - - - - - - - - - - - - - - - - 387

XII.

Mr. Thomas Sandwith's Observations in Medicine and Surgery - - - - - - - - - - - - - - - - 395

XIII.

Dr. Reeder on Diseases of the Heart - - - - - - - - 401

XIV.

Dr. Power on the Female Economy - - - - - - - - 409

XV.

SUPPLEMENTAL REVIEW.

Art. 1. M. Fodéré on Diseases of the Heart - - - - - 417
2. Mr. Mayo on Ulceration of the Cartilages - - - 425
3. Dr. Rizzo's Case of Superfœtation - - - - - - 425
4. Dr. Feron's Case of Neuralgia affecting various Organs - - - - - - - - - - - - - - 426
5. Oil of Croton, a new old Remedy - - - - - - 428
6. M. Pinel on Acute Inflammation of the Medulla Spinalis - - - - - - - - - - - - - - 429
7. Mr. Churchill on Acupuncture - - - - - - - 431
8. Mr. H. Earle on Renal Calculi - - - - - - - 436
9. Colchicum Seeds - - - - - - - - - - - - 437
10. Difficult Parturition - - - - - - - - - - 438
11. Arteritis - - - - - - - - - - - - - - 439
12. Masked Disease of the Brain - - - - - - - - 440
13. Injuries of Bones in Children- - - - - - - 441
14. Secale Cornutum, or Ergot of Rye - - - - - 442
15. Dr. Carson on Vacuity of Arteries - - - - - 443
16. Obscure Uterine Disease - - - - - - - - - 445
17. Ulcer in the Stomach - - - - - - - - - - 446
18. Panckouckerie - - - - - - - - - - - - - 447
19. Ophthalmic Hospital - - - - - - - - - - 448
20. Masked Intermittents - - - - - - - - - - 450
21. Phlebitis - - - - - - - - - - - - - - - 453

XVI.

Extra Limites - - - - - - - - - - - - - - - - 454
Bibliography, Intelligence, Subscribers, &c. &c. - - - - 457

